# Cognitive Improvement and Brain Changes after Real-Time Functional MRI Neurofeedback Training in Healthy Elderly and Prodromal Alzheimer’s Disease

**DOI:** 10.3389/fneur.2017.00384

**Published:** 2017-08-09

**Authors:** Christian Hohenfeld, Nils Nellessen, Imis Dogan, Hanna Kuhn, Christine Müller, Federica Papa, Simon Ketteler, Rainer Goebel, Armin Heinecke, N. Jon Shah, Jörg B. Schulz, Martina Reske, Kathrin Reetz

**Affiliations:** ^1^Department of Neurology, RWTH Aachen University, Aachen, Germany; ^2^Institute of Neuroscience and Medicine (INM-4, 6), Research Centre Jülich GmbH, Jülich, Germany; ^3^JARA-BRAIN Institute Molecular Neuroscience and Neuroimaging, Forschungszentrum Jülich GmbH and RWTH Aachen University, Aachen, Germany; ^4^Department of Cognitive Neuroscience, Maastricht University, Maastricht, Netherlands; ^5^Maastricht Brain Imaging Centre, Maastricht University, Maastricht, Netherlands; ^6^Brain Innovation, Maastricht, Netherlands

**Keywords:** cognitive training, mental imagery, parahippocampus, plasticity, visuospatial memory, neurofeedback, real-time fMRI

## Abstract

**Background:**

Cognitive decline is characteristic for Alzheimer’s disease (AD) and also for healthy ageing. As a proof-of-concept study, we examined whether this decline can be counteracted using real-time fMRI neurofeedback training. Visuospatial memory and the parahippocampal gyrus (PHG) were targeted.

**Methods:**

Sixteen healthy elderly subjects (mean age 63.5 years, SD = 6.663) and 10 patients with prodromal AD (mean age 66.2 years, SD = 8.930) completed the experiment. Four additional healthy subjects formed a sham-feedback condition to validate the paradigm. The protocol spanned five examination days (T1–T5). T1 contained a neuropsychological pre-test, the encoding of a real-world footpath, and an anatomical MRI scan of the brain. T2–T4 included the fMRI neurofeedback training paradigm, in which subjects learned to enhance activation of the left PHG while recalling the path encoded on T1. At T5, the neuropsychological post-test and another anatomical MRI brain scan were performed. The neuropsychological battery included the Montreal Cognitive Assessment (MoCA); the Visual and Verbal Memory Test (VVM); subtests of the Wechsler Memory Scale (WMS); the Visual Patterns Test; and Trail Making Tests (TMT) A and B.

**Results:**

Healthy elderly and patients with prodromal AD showed improved visuospatial memory performance after neurofeedback training. Healthy subjects also performed better in a working-memory task (WMS backward digit-span) and in the MoCA. Both groups were able to elicit parahippocampal activation during training, but no significant changes in brain activation were found over the course of the training. However, Granger-causality-analysis revealed changes in cerebral connectivity over the course of the training, involving the parahippocampus and identifying the precuneus as main driver of activation in both groups. Voxel-based morphometry showed increases in grey matter volumes in the precuneus and frontal cortex. Neither cognitive enhancements, nor parahippocampal activation were found in the control group undergoing sham-feedback.

**Conclusion:**

These findings suggest that cognitive decline, either related to prodromal AD or healthy ageing, could be counteracted using fMRI-based neurofeedback. Future research needs to determine the potential of this method as a treatment tool.

## Introduction

Alzheimer’s disease (AD) is an age-associated neurodegenerative disease. It is the most common type of dementia, but no satisfactory treatment for it has been established yet, posing a major challenge for an ageing society (1, 2).

Alzheimer’s disease is characterised by decline in cognitive function, especially in episodic memory. A domain affected the earliest and severest is visuospatial memory (3–5). Visuospatial memory is associated with the parahippocampal gyrus (PHG) (6), which has been shown to be affected by loss of grey matter (GM) in AD, supposedly related to the visuospatial memory impairment (7, 8). Findings of GM loss are in line with neuropathological changes in early AD (9).

Patterns in cognitive decline, similar to AD can be observed in healthy ageing. While much less severe, healthy ageing is also characterised by cognitive decline (10). Again, visuospatial memory is one of the domains affected the earliest and the most pronounced (11).

An approach to counteract cognitive decline in healthy individuals and patients is cognitive training of specific domains. Successful training regimes have been reported in the literature (12–14). Besides cognitive effects, it was also reported that cognitive training can induce changes in brain structure in the healthy elderly (15) and patients of subjective cognitive impairment (16). Overall, evidence from cognitive training suggests cognitive decline could be at least partially reversible.

During recent years, neurofeedback using real-time functional magnetic resonance imaging (rtfMRI) has become an established method (17). The general idea of neurofeedback is that subjects are provided with information about the state of the brain activation. In the context of fMRI, the activation of one or more regions is usually visualised. Subjects then try to develop strategies to modulate the brain activation and assess the success of these strategies using the provided feedback (18). With the technology available today it has become possible to analyse fMRI data as it is acquired, but general limitations regarding the temporal resolution of fMRI data still apply. Despite its relative novelty, rtfMRI neurofeedback training has already been used in many clinical and non-clinical applications. Research demonstrated improvement of symptoms, e.g., in Parkinson’s disease (19, 20), tinnitus (21), and major depression (22). Two studies on healthy subjects reported improvement of working-memory performance after rtfMRI neurofeedback training of the dorsolateral prefrontal cortex (23, 24). In addition to these findings, a study employing electroencephalography (EEG)-based neurofeedback training combined with diffusion tensor imaging of the brain, showed that structural brain changes could be induced (25).

Due to the results reported in cognitive training studies regarding cognitive decline and due to the success of neurofeedback applications in many diseases, the present study applied rtfMRI neurofeedback training to prodromal AD (pAD) patients (26) and the healthy elderly. Due to their crucial roles in AD and healthy ageing, we chose visuospatial memory and the PHG as training targets. Subjects would actively recall visuospatial memories while simultaneously trying to increase PHG activation.

We hypothesised that the training paradigm would enhance visuospatial memory performance and also induce changes in brain structure and function in regions related to visuospatial memory, mental imagery, and cognitive control.

## Materials and Methods

### Participants

30 subjects completed the study. Subjects were divided into three groups: healthy elderly experimental group (HC, *n* = 16), healthy elderly sham-feedback group serving as a control group to verify whether feedback of PHG activation is required (SH, *n* = 4) and patients of pAD (PA, *n* = 10). Inclusion criteria were age between 50 and 80 years; right-handedness as determined by a simplified version of the Edinburgh Handedness Inventory (27); native language German; and the ability to provide written informed consent. Exclusion criteria included a history of neurologic or psychiatric disorders (except for pAD in group PA); concurrent use of psychotropic medication; MRI contraindications; familiarity with the Research Centre Jülich campus (see below); colour-blindness.

Healthy subjects were recruited in the RWTH Aachen university hospital and its vicinity. Patients of pAD were referred to the study from the memory clinic of the neurological department of RWTH Aachen University after being diagnosed according to the relevant criteria (26). AD diagnoses were based on a clinical and standardised neuropsychological test battery (CERAD) (28), MRI and cerebrospinal fluid-related neurodegeneration markers: amyloid β_1-42_, amyloid β_1-40_, amyloid β_1-42_/β_1-40_ ratio, total tau, and phospho-tau.

After full introduction of the study, all subjects provided written informed consent for participation. The local institutional review board at RWTH Aachen University approved the experiment and its procedures in accordance with the declaration of Helsinki (29).

### Study Design and Protocol

The study protocol consisted of five examination days (T1–T5), with a time interval of 2–7 days in between. The interval was chosen based on previous studies, but also mandated by constraints in MRI scanner availability. Due to scheduling problems, this interval was exceeded in some cases: in group HC once in five subjects and twice in two subjects (mean = 9.904, SD = 18.392, median = 3); in group PA once in six subjects (mean = 7.05, SD = 9.438, median = 5); in group SH once in one subject (mean = 4.313, SD = 2.358, median = 5).

**Figure 1:**
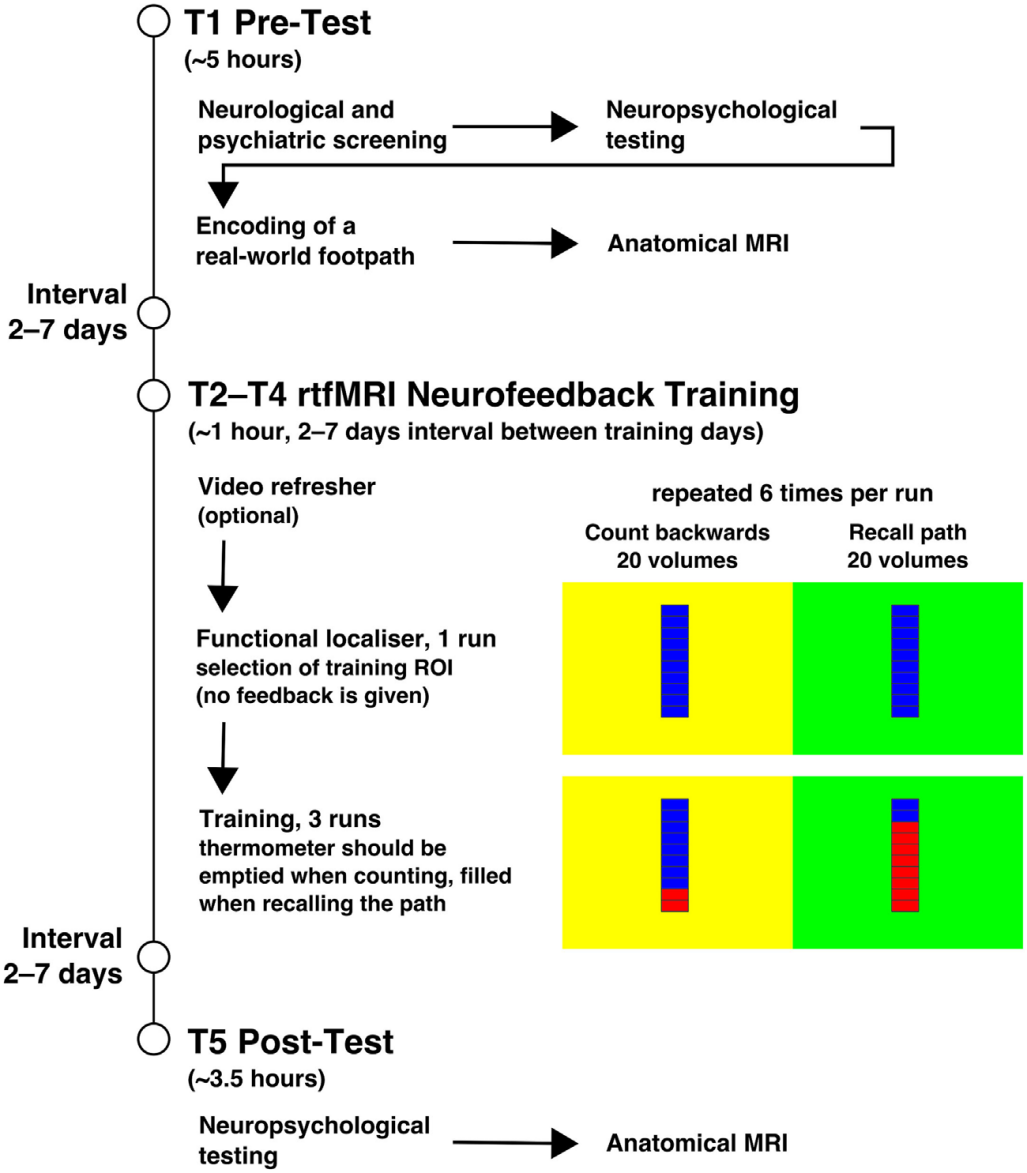
Procedures. The figure summarises the procedures of the experiment. The experiment started with the pre-test on T1 and the illustrated procedure. T2–T4 included the neurofeedback training, which is displayed with more detail. Each training session started with the functional localiser in which the feedback stimulus was shown, but no feedback was given. It was used to select a region of interest (ROI) in the left parahippocampus as feedback source for the training. Feedback was shown during the three subsequent training runs. The background colour of the stimulus indicated the task with it being counting backwards at yellow and recalling the path from T1 at green. After the three training days, at T5 the post-test took place. The interval between all training days was usually 2–7 days.

A quasi-experimental design consisting of pre-test (T1), intervention (T2–T4), and post-test (T5) was employed. At T1, a neurological and neuropsychological examination, the visuo-spatial memory task of encoding a real-world footpath, and an anatomical MRI scan of the brain were performed. At T2–T4, rtfMRI neurofeedback training took place, in which the subjects were asked to recall the footpath encoded in the visuospatial memory task (see below for details). This was followed by a neuropsychological examination and another anatomical scan of the brain at T5. All examinations were carried out at the Research Centre Jülich, Germany. The procedures are summarised in Figure 1.

#### Neurological, Psychopathological, and Neuropsychological Examination

Trained professionals rated the neurological health and cognitive abilities of subjects. Neurological symptoms with a focus on memory-related problems were queried in a semi-structured interview and all participants received a basic neurological examination. Subjects were assessed using the SKID I (30) screening questions, a structured interview for diagnosis of psychiatric disorders from DSM-IV axis I (31). Also, subjects filled in the Beck Depression Inventory II (BDI-II) (32) to quantify self-experienced symptoms of depression.

Tests of the neuropsychological battery were selected based on the following criteria: (1) visuospatial memory should be represented; (2) a broad range of cognitive abilities should be metered so potential generalisation effects could be observed; (3) due to repeated testing availability of parallel test versions implementing the same task with different contents was important. The test battery comprised of the following tests: the Visual and Verbal Memory Test (VVM) (33) on visuospatial and verbal memory, with immediate and delayed recall conditions; the subtests on visual and verbal memory both with immediate and delayed recall, as well as the forward and backward digit-span tasks of the revised Wechsler Memory Scale (34) to assess short-term memory and working-memory capabilities. For a short assessment of overall cognitive performance, the Montreal Cognitive Assessment (MoCA) (35, 36) was used. From the CERAD Plus (28), Trail Making Tests A and B (TMT-A and TMT-B), which test cognitive processing speed (TMT-A and -B) and task switching ability (TMT-B) were drawn on. The Visual Patterns Test (VPT) (37), which measures the capacity of visual working memory was further included into the test battery. Only at T1, the Multiple Choice Word Test (MWT-B) (38) as an estimate of premorbid intelligence was administered.

#### Visuospatial Memory Task: Memorising a Real-World Footpath

At T1, subjects encoded one out of three predefined real-world footpaths (“central,” “west,” “north”) on the campus of the Research Centre Jülich. Paths were randomly assigned to subjects. From group HC, five subjects learned path central, five west, and six north. From group PA, three subjects learned path central, six west, and one north. From group SH, two subjects learned path central, another two subjects learned path west.

The instruction was to memorise as many details of the path as possible while the examiner guided the subject along the path. A stop was made at 10 waypoints (prominent landmarks, usually buildings with unique features). At each stop, subjects were informed about the name of the waypoint and they were asked to specifically memorise the waypoint. As soon as subjects indicated that they were certain that they had memorised the waypoint, the tour was continued.

Paths were between 1.5 and 2 km long, and a single tour was usually completed within 30 min. Paths were mostly exclusive with only few crossing sections. At the beginning of each of the following sessions, subjects could watch a 3-min fast-forward video of the learned path to assure sufficient memory.

### Brain Imaging

Brain imaging was performed on a Siemens (Erlangen, Germany) MAGNETOM Trio whole-body 3T MR scanner using standard gradients and a 32-channel phased-array head coil for signal reception; the body coil was used for radiofrequency transmission. Participants were positioned head-first supine. Foam padding was used within the head coil to limit movement. An MR-compatible mirror system was attached to the coil so that subjects could look out to the back of the scanner where an MR-compatible LED screen was mounted.

#### Imaging Parameters

On all examination days, high-resolution anatomical scans using a magnetisation-prepared rapid gradient echo (MP-RAGE) sequence were acquired (TR = 2,250 ms, TE = 3.03 ms, flip angle = 9°, slice thickness = 1 mm, 256 × 256 in-plane matrix, acquisition time: 5 min 14 s). For rtfMRI neurofeedback and functional localiser runs, an echo planar imaging sequence was used (TR = 2,000 ms, TE = 62 ms, flip angle = 79°, field-of-view = 192 × 192, 64 × 64 in-plane matrix, 32 slices with 3 mm thickness and an inter-slice-gap of 1.2 mm, 240 whole-brain volumes, acquisition time: 8 min).

#### Neurofeedback Training: Setup and Implementation

On T2–T4, the neurofeedback training was performed. Following the anatomical scan, a functional localiser run and three neurofeedback training runs were carried out. MRI data were transferred by network from the scanner console to an additional computer for real-time analysis. Images were analysed using Turbo-BrainVoyager (Brain Innovation, Maastricht, The Netherlands) and the analysis result (a relative numerical value of activation) was written to disk and read by a Python (https://www.python.org/) script within PsychoPy (39) to generate the visual feedback stimulus.

**Figure 2:**
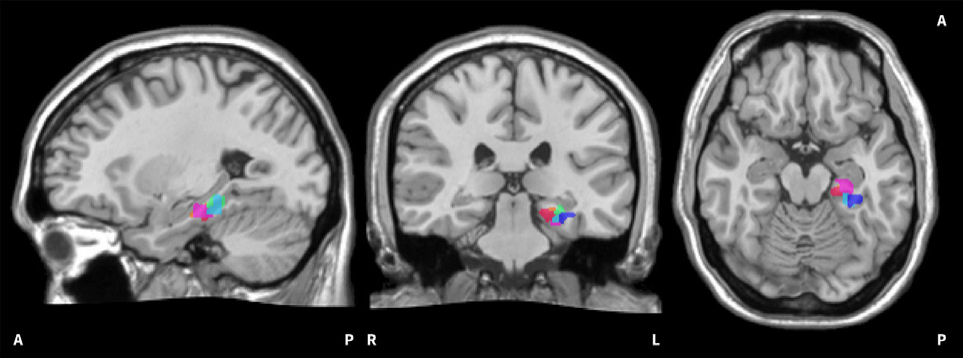
Visualisation of on-line regions of interest (ROIs). The image shows all on-line ROIs from two exemplary subjects. The red/pink hues belong to the first subject, the blue/green hues to the second subject. It can be seen that the selected ROIs in the left parahippocampus are scattered to a certain extent. The ROIs are superimposed onto the Colin 27 average brain. Copyright© 1993–2009 Louis Collins, McConnell Brain Imaging Centre, Montreal Neurological Institute, McGill University.

The functional localiser of each training day enabled the experimenter to select a region of interest (ROI) for the subsequent feedback runs using an overlay of functional results on anatomical data. For groups HC and PA, a ROI within the left PHG was selected as training target. For group SH, a region from the left primary somatosensory cortex was chosen. The ROI had a size of 500–700 voxels (vx) and was selected at a statistical threshold of *t* ≥ 3 in HC and PA; for SH the activation threshold was lowered as far as required to select a ROI in the desired region. ROIs from two subjects are visualised in Figure 2. Brain activation derived from the selected ROI was visualised as a thermometer bar during neurofeedback training runs (Figure 1). The task during the functional localiser was the same as in the subsequent training runs (see below), but no visual feedback was provided.

During training runs, baseline phases and upregulation phases (six each) constantly alternated, lasting 20 whole-brain acquisitions (40 s) each. Phases were indicated by the background colour of the visual feedback stimulus, with yellow indicating baseline phases and green indicating upregulation phases. Upregulation phases required subjects to actively remember and imagine the path they had learned at T1 to try enhancing brain activation visualised in the thermometer bar. Baseline phases required subjects to slowly count backwards from 100 to bring down the thermometer bar. The baseline condition was designed to be very different from the spatial navigation task in the upregulation phases and thus to elicit different patterns of brain activation. Each rtfMRI neurofeedback session lasted about 40–45 min.

#### Debriefing after rtfMRI Runs

On each training day, participants were debriefed after the functional localiser and after each of the three feedback runs to ensure that all subjects followed the given instructions. After the functional localiser, subjects were asked whether they had been able to recall the learned path well (yes or no). Further, subjects were asked what they had done when the background of the thermometer was yellow and green, respectively. Last, subjects were asked whether they felt that they were able to influence the thermometer (yes/partially/no).

### Statistical Analysis

#### Behavioural Analysis

Neuropsychological test scores were transformed into percent ranks according to normative data provided with the tests’ manuals, stratified by age, education and/or gender (WMS: age; VVM and VPT: age and education; TMT-A/B: age, education, gender). For the MoCA, raw scores were used and for the MWT-B an estimated premorbid intelligence quotient was generated.

Data were analysed using R (40). For each neuropsychological test a linear mixed model was calculated, using examination time point as within-subject predictor and if the normative data for the specific test did not already correct for it gender and/or age as additional predictors. To assess model fit $\Omega_{0}^{2}$ is reported (41). Furthermore age, BDI-II score, and MWT-B score were tested for differences between groups using an ANOVA. The significance threshold was set to α = 0.05.

If a data point from a subject in a neuropsychological test was missing, that data point was excluded from the respective analysis. This was true for two subjects from the group HC, with data missing due to technical problems for the WMS verbal memory task: in one case on T1 and T5, respectively.

#### fMRI Analysis

The fMRI data gathered during training sessions was analysed using the software BrainVoyager QX, the NeuroElf toolbox (http://neuroelf.net/) for Matlab (The MathWorks, Natick, MA, USA) and R to extract and analyse time course data.

Functional data were pre-processed using Brain Voyager QX, employing slice scan time correction, three-dimensional motion correction and high-pass filtering using Fourier analysis (cutoff of two sine and cosine cycles over the course of the data). Additionally, spatial smoothing with a Gaussian kernel of 6 mm full-width at half-maximum (FWHM) was conducted. Anatomical data were skull stripped, intensity inhomogeneity corrected, and transformed to Talairach space. Functional and anatomical data were then co-registered and combined into a single dataset.

To assess brain activation, a general linear model (GLM) with a single predictor of upregulation and movement parameters as covariates was computed with Brain Voyager QX for each neurofeedback run within a group. Brain activation was assessed at uncorrected *p* < 0.001 and at a threshold of Bonferroni corrected *p* < 0.05. Minimum cluster size was determined using Alphasim (42) within Neuroelf. To identify brain regions active during all neurofeedback training runs, data were then averaged across all nine neurofeedback runs and a table of active clusters was generated.

Using R, the course of activation was extracted from the pre-processed fMRI data for a cluster of the left PHG as determined by the GLM. Data were averaged over the entire cluster per run and subject in a first step and then the percent signal change (PSC) was calculated. Here, the second half of each baseline phase was used as reference for each subsequent upregulation phase and the difference expressed as PSC. The PSC for upregulation was averaged per run, leading to nine PSC values per subject. An analysis of variance was then calculated with the PSC as dependent measure and the training run as within-subjects factor. As measure of effect size generalised eta squared ($\eta_{G}^{2}$) is reported (43).

As one subject in the healthy control-group aborted the measurement on T3, only one neurofeedback run is present for this subject at that specific time point. Therefore, this subject was excluded from the PSC analysis.

#### Granger-Causality-Analysis (GCA)

To assess cerebral connectivity, GCA was performed in R. GCA is an approach to assess effective connectivity (44). Its principle is that given two time series *X* and *Y, Y* is said to g-cause *X* if the future of X is predicted better incorporating information from *Y* than from *X* alone.

Clusters to be used for a pairwise GCA were selected *post hoc* based on functional results of groups HC and PA, so that all active regions were part of the analysis. In case a region was active in both groups or multiple clusters of activation were present within one region, only the largest cluster was selected. Clusters below 20 functional voxels in size were not considered for selection. Raw activation time courses were extracted and averaged per training day for each cluster and group. Data were then checked for unit-roots and autocorrelations as well as time courses with high colinearity. The Bayesian Information Criterion was determined to select the lag size for the analysis. Finally, a F-test for Granger-causality was computed for each pairwise combination of entered clusters and p-values were adjusted using the Holm method.

#### Voxel-Based Morphometry (VBM)

Voxel-based morphometry of structural MRI data were performed using the CAT12 toolbox (45), implemented in SPM12 (http://www.fil.ion.ucl.ac.uk/spm/) running on Matlab. As we were interested in GM volume changes from T1 to T5, we used the CAT12 protocol for processing of longitudinal data. Pre-processing steps incorporated intra-subject realignment, bias correction, segmentation, and spatial normalisation. After an initial realignment of individual anatomical scans to standardised (MNI) space, the mean of the realigned images was calculated and used as reference image in a subsequent realignment. The realigned individual images were then bias-corrected to account for signal inhomogeneities. The mean image was segmented into GM, white matter and cerebrospinal fluid and normalised using DARTEL. The resulting spatial normalisation parameters were then applied to the segmentations of the bias-corrected individual images of both time points, which were again realigned. To allow comparison of the absolute tissue volume, voxel values were modulated using the Jacobian determinants (i.e., linear and non-linear components) derived from the spatial normalisation. Finally, the modulated GM images were smoothed with a Gaussian kernel of 8 mm FWHM. Between-group differences in GM volumes at baseline were assessed using two-sample *t*-tests adjusted for total intracranial volume (TIV), age, and gender. In a flexible factorial design with the factors “subject,” “group,” and “time” and after including TIV as nuisance parameter, main effects of time and group-by-time interactions were tested. To avoid possible edge effects around the border between tissue types, an absolute GM threshold of 0.01 was applied. For voxel-wise statistical analysis, we used a cluster-level family wise error correction of *p* < 0.05 across the whole-brain (uncorrected height threshold of *p* < 0.001) adjusted for non-stationarity (46). Additionally, PHG and hippocampal volumes were derived as ROI, corrected for TIV and compared between groups.

#### Debriefings

The data from debriefings after rtfMRI training runs were first converted into numerical scores. For the question whether subjects thought that they were able to voluntarily influence the thermometer bar, the answer no was converted to 0, partially to 1 and yes to 2 (leading to a maximum total score of 6 per training day). As equidistance between answers could not be assumed, only statistics requiring at most ordinal scale were applied to debriefing data. To assess changes in debriefing response over the course of the training, a Friedman test over all three training days was computed for each experimental group. For analysis of differences between groups Kruskal–Wallis tests were performed.

## Results

### Sample

In total, 10 female and 20 male subjects completed the study (mean age 64.567, SD = 7.838). Of these, seven female and nine male subjects were part of group HC; two female and eight male subjects formed group PA; and one female and three male subjects were part of group SH. There were no differences between groups in age [*F*(2,27) = 0.350, *p* = 0.709, η^2^ = 0.025] or BDI-II score [*F*(2,27) = 0.655, *p* = 0.528, η^2^ = 0.044]. There was a difference in estimated premorbid intelligence between groups [*F*(2,27) = 5.079, *p* = 0.013, η^2^ = 0.273], where pairwise *t*-tests suggest that estimated premorbid intelligence was bigger in HC compared to PA, but no difference between SH and both other groups was present. See Table 1 for full demographic details and neurodegeneration markers in group PA.

**Table 1:** Demographics and characteristics of the sample.

	HC	PA	SH
*n*	16	10	4
Female	7	2	1
Male	9	8	3
Mean age (±SD; range)	63.5 (±6.663; 53–76)	66.2 (±8.930; 53–80)	64.75 (±9.453; 51–73)

**Education**			

Higher education	7	3	2
13 years in school	4	2	1
12 years in school	2	0	0
10 years in school	3	2	0
9 years in school	0	2	0
8 years in school	0	1	1

**Psychometrics**			

Mean BDI score (±SD; range)	3.313 (±3.807; 0–11)	5.2 (±5.203; 0–15)	6 (±9.416; 0–20)
Mean MWT-B IQ (±SD; range)	124 (±9.295; 112–136)	107.8 (±14.062; 91–130)	121.5 (±21.734; 100–143)

**Neurodegeneration markers in cerebrospinal fluid (mean ± SD; range)**			

Amyloid β_1-42_ (pg/ml)	–	597.111 (±163.164; 409–928)	–
Amyloid β_1-40_ (pg/ml)	–	14,971.125 (±6,008.380; 4,051–25,356)	–
β_1-42_/β_1-40_ ratio	–	0.476 (±0.243; 0.24–1)	–
Total tau (pg/ml)	–	367.444 (±255.557; 95–927)	–
Phospho-tau (pg/ml)	–	74.556 (±35.606; 28–126)	–

*An overview of certain characteristics of participants is given. Cerebrospinal fluid characteristics were unavailable for one patient, the amyloid β_1-40_ value was missing in one additional case. Reference values were used from the Neurochemical Laboratory at the University of Göttingen, Germany: β-amyloid_1-42_: >450 pg/ml; β-amyloid ratio [(β-amyloid_1-42_/β-amyloid_1-40_) × 10] >0.5; total tau protein: <450 pg/ml; phospho-tau protein_181p_: >61 pg/ml*.

*BDI, Beck Depression Inventory II; MWT-B, Multiple-Select-Word-Choice-Test B; IQ, estimated premorbid intelligence quotient*.

### Neuropsychological Baseline Profile and Changes after rtfMRI Neurofeedback Training

In groups HC and SH, average neuropsychological scores were in the normal range in both the pre- (T1) and post-test (T5). In group PA scores of the following tests were below the normal range: MoCA, VVM Verbal Memory immediate recall (T1 only) and delayed recall, WMS Visual Memory delayed recall (T5 only), WMS Verbal Memory delayed recall and TMT-B (see Table 2 for details).

**Table 2:** Descriptive statistics.

Test	HC		PA		SH	
	Pre	Post	Pre	Post	Pre	Post
MoCA	26.813 ± 1.974	28 ± 1.592	24.8 ± 3.225	24.5 ± 2.915	26 ± 4.243	26.25 ± 3.775
Visual and Verbal Memory Test (VVM) Visp 1	53.188 ± 24.460	69.063 ± 23.268	38.2 ± 35.080	60.2 ± 20.778	79.5 ± 19.140	79 ± 16.513
VVM Visp 2	50.563 ± 25.222	59.5 ± 22.675	24.2 ± 23.794	48.7 ± 28.987	59.25 ± 24.541	66.25 ± 9.639
VVM Verbal 1	49.063 ± 26.941	51.25 ± 28.252	17.6 ± 22.887	19.4 ± 15.551	67 ± 13.589	33 ± 42.166
VVM Verbal 2	46.063 ± 25.878	47.938 ± 30.163	16.6 ± 24.496	14.6 ± 15.472	56.5 ± 21	36 ± 31.294
Wechsler Memory Scale (WMS) Visual 1	50.5 ± 32.922	61.75 ± 31.298	37.6 ± 36.855	18.2 ± 17.781	73.75 ± 23.614	64.25 ± 27.945
WMS Visual 2	55.125 ± 29.209	53.750 ± 31.792	24.6 ± 31.224	13.4 ± 15.072	63 ± 38.730	49.25 ± 31.170
WMS Verbal 1	52.933 ± 29.550	62.2 ± 26.474	19.9 ± 23.727	25.9 ± 25.736	44.75 ± 38.448	51.75 ± 34.760
WMS Verbal 2	54.733 ± 32.017	56.6 ± 30.016	14.7 ± 21.145	16 ± 21.566	42.25 ± 29.216	31.25 ± 37.277
WMS Digit Fw	77.5 ± 16.653	71.313 ± 23.105	49.2 ± 32.454	42.8 ± 35.496	59 ± 25.179	78.5 ± 17.059
WMS Digit Bw	59.25 ± 29.051	72.875 ± 20.720	45.8 ± 35.443	58.4 ± 27.893	49.5 ± 27.574	61.5 ± 17.673
TMT-A	46.366 ± 9.132	48.504 ± 10.885	45.554 ± 19.283	48.974 ± 19.215	60.230 ± 7.366	57.673 ± 9.794
TMT-B	19.596 ± 14.509	19.464 ± 13.058	15.424 ± 20.475	14.952 ± 16.938	37.5 ± 25.613	32.098 ± 23.357
Visual Patterns Test	60.625 ± 30.434	62.813 ± 29.324	44 ± 31.605	46 ± 21.211	53.75 ± 33.260	73.75 ± 17.5

*Given is mean ± 1 SD for each group at each time point. Scores are percent ranks, except the MoCA for which raw scores are shown*.

*Visp, visuospatial; fw, forward; bw, backward*.

*Immediate recall is denoted by 1, delayed recall by 2*.

In HC, increases in scores from pre to post-test were found for the visuospatial task of the VVM in the immediate recall condition [*t*(15) = −2.690, *p* = 0.017], for the MoCA [*t*(15) = −3.721, *p* = 0.002] and the backward digit-span task of the WMS [*t*(15) = −2.817, *p* = 0.013]. Additionally, a trend was found for an increase of scores in the delayed recall condition of the visuospatial memory task of the VVM [*t*(15) = −1.864, *p* = 0.082].

Group PA displayed an increase in scores for the delayed recall of the visuospatial memory task of the VVM [*t*(9) = −3.041, *p* = 0.014]. A trend for an increase of scores in the immediate recall of that task [*t*(9) = −2.187, *p* = 0.057] and a further trend for a decrease in scores for immediate recall condition of the WMS visual memory task [*t*(9) = 2.188, *p* = 0.056] were present.

No changes in scores from pre to post-test were found in SH. An overview of all models is given in Table 3.

**Table 3:** Linear mixed model results for examination time point.

Test	HC	PA	SH
Visual and Verbal Memory Test (VVM) Visp 1	*t* = −2.690, *p* = 0.017, ${\text{Ω}}_{0}^{2}$ = 0.679	*t* = −2.187, *p* = 0.057, ${\text{Ω}}_{0}^{2}$ = 0.642	*t* = 0.044, *p* = 0.968, ${\text{Ω}}_{0}^{2}$ = 0.337
VVM Visp 2	*t* = −1.864, *p* = 0.082, ${\text{Ω}}_{0}^{2}$ = 0.809	*t* = −3.041, *p* = 0.014, ${\text{Ω}}_{0}^{2}$ = 0.768	*t* = −0.780, *p* = 0.492, ${\text{Ω}}_{0}^{2}$ = 0.732
VVM Verbal 1	*t* = −0.353, *p* = 0.729, ${\text{Ω}}_{0}^{2}$ = 0.733	*t* = −0.214, *p* = 0.835, ${\text{Ω}}_{0}^{2}$ = 0.228	*t* = 1.441, *p* = 0.245, ${\text{Ω}}_{0}^{2}$ = 0.321
VVM Verbal 2	*t* = −0.419, *p* = 0.681, ${\text{Ω}}_{0}^{2}$ = 0.887	*t* = 0.262, *p* = 0.799, ${\text{Ω}}_{0}^{2}$ = 0.504	*t* = 1.945, *p* = 0.318, ${\text{Ω}}_{0}^{2}$ = 0.515
MoCA	*t* = −3.721, *p* = 0.002, ${\text{Ω}}_{0}^{2}$ = 0.873	*t* = 1.152, *p* = 0.279, ${\text{Ω}}_{0}^{2}$ = 0.982	*t* = −0.333, *p* = 0.761, ${\text{Ω}}_{0}^{2}$ = 0.964
Wechsler Memory Scale (WMS) Verbal 1	*t* = −1.197, *p* = 0.252, ${\text{Ω}}_{0}^{2}$ = 0.866	*t* = −0.708, *p* = 0.497, ${\text{Ω}}_{0}^{2}$ = 0.616	*t* = −0.392, *p* = 0.721, ${\text{Ω}}_{0}^{2}$ = 0.609
WMS Verbal 2	*t* = −0.066, *p* = 0.949, ${\text{Ω}}_{0}^{2}$ = 0.868	*t* = −0.194, *p* = 0.850, ${\text{Ω}}_{0}^{2}$ = 0.685	*t* = 0.522, *p* = 0.638, ${\text{Ω}}_{0}^{2}$ = 0.362
WMS Visual 1	*t* = −1.430, *p* = 0.173, ${\text{Ω}}_{0}^{2}$ = 0.699	*t* = 2.187, *p* = 0.056, ${\text{Ω}}_{0}^{2}$ = 0.735	*t* = 0.713, *p* = 0.528, ${\text{Ω}}_{0}^{2}$ = 0.577
WMS Visual 2	*t* = 0.257, *p* = 0.800, ${\text{Ω}}_{0}^{2}$ = 0.862	*t* = 1.450, *p* = 0.181, ${\text{Ω}}_{0}^{2}$ = 0.701	*t* = 1.794, *p* = 0.171, ${\text{Ω}}_{0}^{2}$ = 0.950
WMS Digit Fw	*t* = 1.655, *p* = 0.119, ${\text{Ω}}_{0}^{2}$ = 0.831	*t* = 0.523, *p* = 0.614, ${\text{Ω}}_{0}^{2}$ = 0.557	*t* = −2.832, *p* = 0.066, ${\text{Ω}}_{0}^{2}$ = 0.891
WMS Digit Bw	*t* = −2.817, *p* = 0.013, ${\text{Ω}}_{0}^{2}$ = 0.841	*t* = −1.667, *p* = 0.130, ${\text{Ω}}_{0}^{2}$ = 0.848	*t* = −1.260, *p* = 0.297, ${\text{Ω}}_{0}^{2}$ = 0.831
Visual Patterns Test	*t* = −0.353, *p* = 0.730, ${\text{Ω}}_{0}^{2}$ = 0.787	*t* = −0.263, *p* = 0.799, ${\text{Ω}}_{0}^{2}$ = 0.770	*t* = −1.414, *p* = 0.252, ${\text{Ω}}_{0}^{2}$ = 0.701
TMT-A	*t* = −0.781, *p* = 0.447, ${\text{Ω}}_{0}^{2}$ = 0.583	*t* = −1.022, *p* = 0.333, ${\text{Ω}}_{0}^{2}$ = 0.919	*t* = 0.502, *p* = 0.650, ${\text{Ω}}_{0}^{2}$ = 0.488
TMT-B	*t* = 0.034, *p* = 0.974, ${\text{Ω}}_{0}^{2}$ = 0.518	*t* = 0.257, *p* = 0.802, ${\text{Ω}}_{0}^{2}$ = 0.976	*t* = 0.514, *p* = 0.626, ${\text{Ω}}_{0}^{2}$ = 0.805

*Shown are results for differences in examination time points for all neuropsychological tests in all groups. Additionally the overall model fit is given. Models may include the additional predictors gender (all except for TMT-A/B) and age (all WMS subtests), where the normative data did not respect these variables (data not shown)*.

*Visp, visuospatial memory; fw, forward; bw, backward*.

*1, immediate recall; 2, delayed recall*.

### Brain Activation during Training

**Figure 3:**
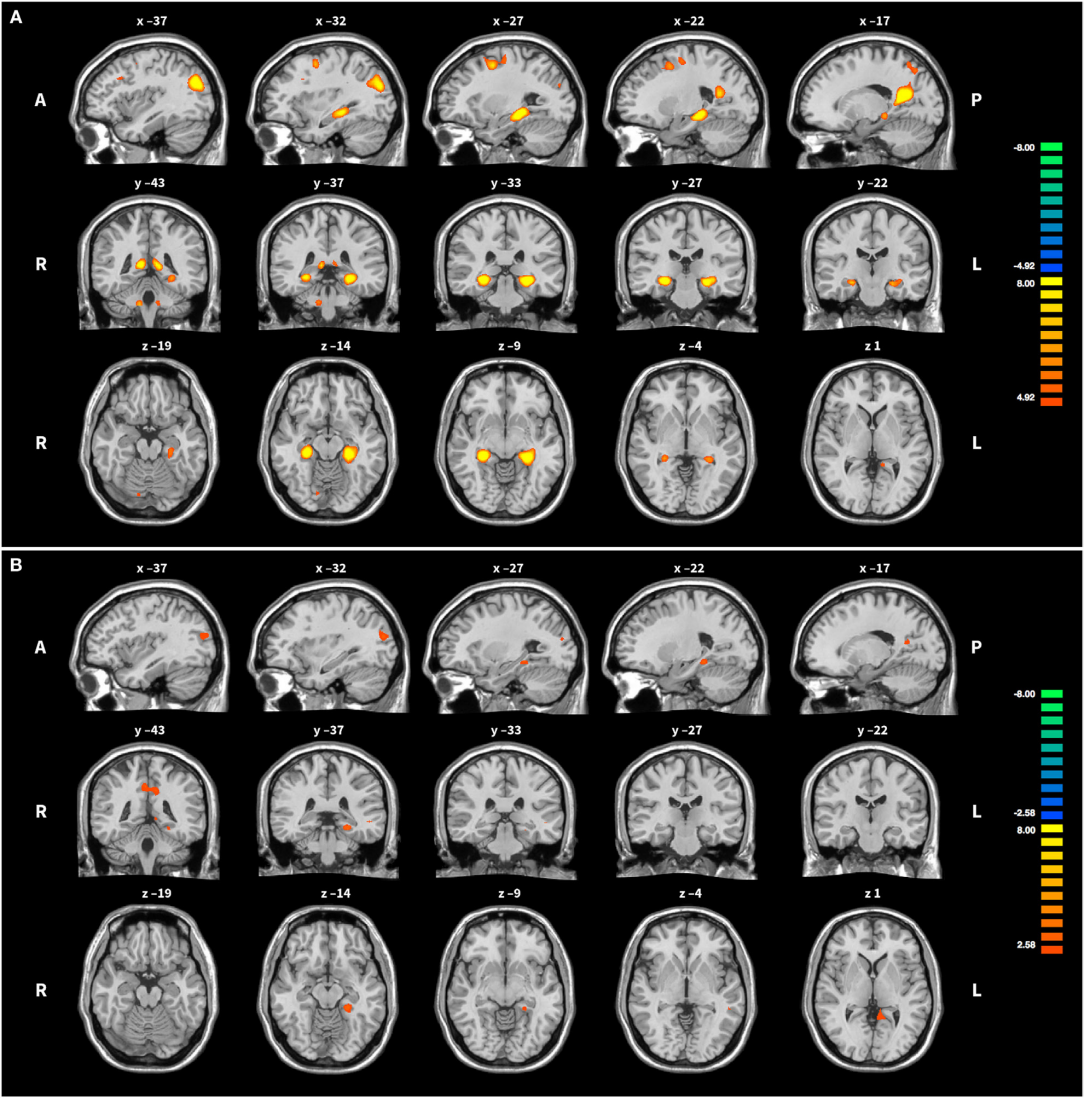
Activation during upregulation. **(A)** Group HC: the middle column shows the peak of activation in the left parahippocampal gyrus, the target region of the training. Some further activation in frontal, parietal and cerebellar areas is visible as well. **(B)** Group PA: for easier comparison slices shown are the same as for group HC. Note the more liberal activation threshold in group PA. For both groups activation was averaged across all training runs and superimposed onto the Colin 27 average brain. Copyright (c) 1993–2009 Louis Collins, McConnell Brain Imaging Centre, Montreal Neurological Institute, McGill University.

In groups HC and PA, activation of the target region (left PHG) was revealed, but not in SH (see Table 4 and Figure 3). In HC, activation was further found in the right precuneus, left posterior cingulate, right PHG, bilaterally in the superior occipital gyrus and middle frontal gyrus, as well as in cerebellar areas. Averaged over the course of the training, in group PA activation was only found in the left precuneus and right posterior cingulate. A small cluster within the left PHG is below cluster-size threshold. On T3 only, broader activation was found including the left precuneus, bilateral middle frontal gyrus, left medial frontal gyrus, right cerebellar tonsil, left precentral gyrus, left middle occipital gyrus and left parahippocampus (see Table 5).

**Table 4:** Brain activation averaged across all neurofeedback runs.

Region	BA	*k*	*x*	*y*	*z*	*T*_max_	*T*_mean_
**Group HC, *p* < 10^−6^, *k* > 2, df = 3,727**							
R Precuneus	31, 7	855	16	−62	18	15.126	7.323
L Parahippocampal gyrus (PHG)		150	−27	−33	5	12.086	7.172
R PHG		101	27	−31	4	11.882	7.397
L Superior occipital gyrus	19	164	−37	−75	23	10.767	6.948
R Superior occipital gyrus	39	105	39	−73	20	10.429	6.518
L Middle frontal gyrus	6	110	−29	−2	46	8.077	5.748
R Cerebellar anterior lobe		20	11	−40	−27	6.984	5.813
R Middle frontal gyrus	6	32	27	−6	48	6.764	5.571
R Declive		11	15	−62	−18	6.024	5.294
R Declive		16	15	−80	−11	6.019	5.357
R Cerebellar tonsil		20	37	−54	−32	5.739	5.269
L Middle Frontal gyrus	9	12	−40	17	31	5.695	5.252
L Cerebellar anterior lobe		9	−7	−40	−25	5.647	5.229
R Culmen		10	30	−51	−21	5.480	5.177
L Medial frontal gyrus	6	2	−11	2	56	5.251	5.082
L Cingulate gyrus	31	4	−16	−40	34	5.208	5.185
L Middle frontal gyrus	47	4	−35	38	−2	−5.452	−5.377
R Precentral gyrus	4	3	54	−13	40	−5.835	−5.221
**Group PA, *p* < 0.001, *k* > 16, df = 2,329**							
L Posterior cingulate	29	25	12	−50	13	4.644	3.806
L Precuneus	7	31	−3	−60	36	3.784	3.582
**Group SH, *p* < 0.001, *k* > 23, df = 931**							
None							

*A contrast for upregulation > baseline was computed for each run and then data were averaged across all runs for each group. Size thresholds were determined using Alphasim. BA, Brodmann area; k, cluster size (3 mm^3^ voxels); *x*/*y*/*z*, peak coordinate in Talairach space; *T*_max_ and *T*_mean_, peak and average *T-*value of the cluster; df, degrees of freedom*.

**Table 5:** Brain activation during neurofeedback on T3 in group PA.

Region	BA	*k*	*x*	*y*	*z*	*T*_max_	*T*_mean_
L Precuneus	7	487	0	−57	38	5.491	3.990
L Medial Frontal Gyrus	8	53	−12	28	38	5.018	3.788
L Precentral Gyrus	6	37	−47	4	34	4.776	3.770
L Parahippocampal Gyrus		24	−27	−36	5	4.607	3.849
R Middle Frontal Gyrus	9	40	43	15	29	4.409	3.667
L Superior Temporal Gyrus	39	23	−48	−56	21	4.398	3.579
L Middle Occipital Gyrus	19	28	−31	−83	16	4.230	3.686
R Cerebellar Tonsil		36	25	−65	−32	4.176	3.555
L Middle Frontal Gyrus	6	32	−36	14	43	4.059	3.662
R Middle Occipital Gyrus	19	22	40	−72	11	4.032	3.559
L Middle Temporal Gyrus	21	22	−53	−44	6	3.921	3.553
L Middle Frontal Gyrus	6	17	−27	1	48	3.802	3.486

*As a peak in activation was present on T3 more activation is found here during upregulation phases than on the other two training days. Data are thresholded to *p* < 0.001 and *k* > 16; df = 2,329. BA, Brodmann area; k, cluster size (3 mm^3^ voxels); *x*/*y*/*z*, peak coordinate in Talairach space; *T*_max_ and *T*_mean_, peak, and average T-value of the cluster; df, degrees of freedom*.

**Figure 4:**
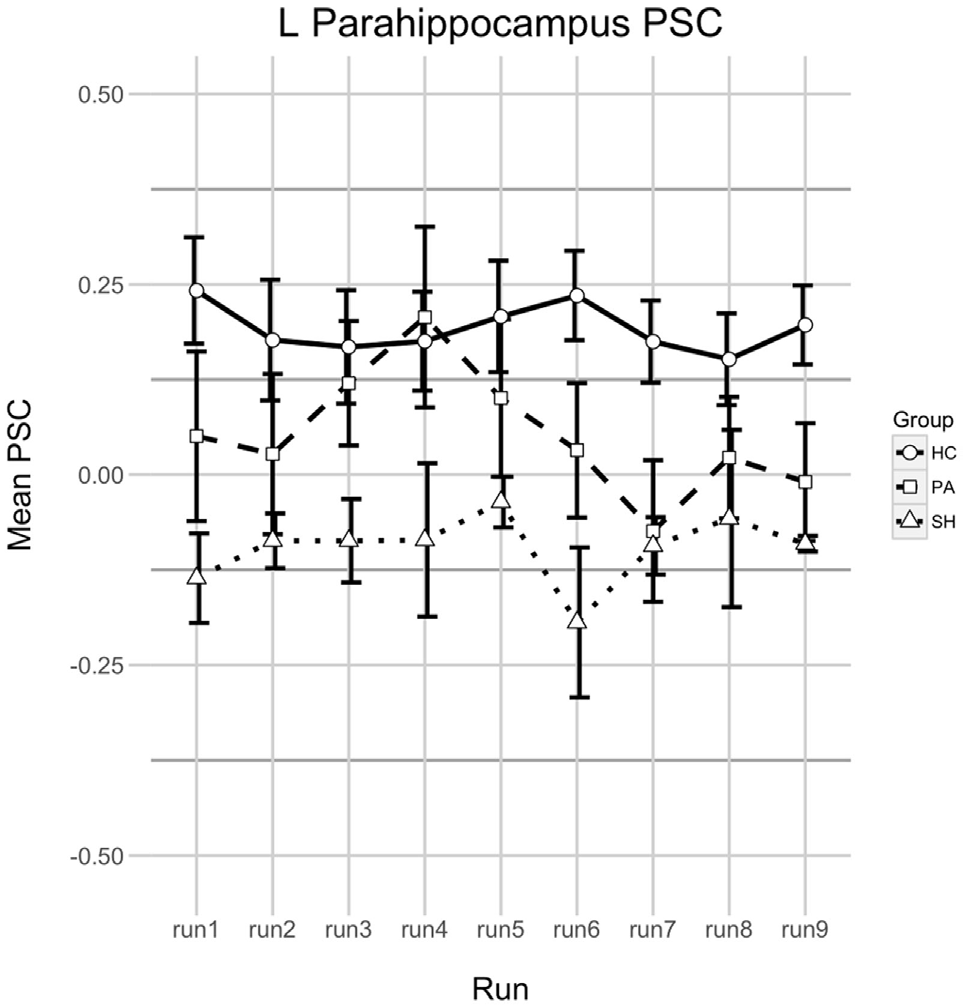
Percent signal change (PSC) in the left parahippocampus during upregulation. Shown is the PSC of upregulation phases in all real-time functional magnetic resonance imaging neurofeedback training runs. A positive value means that parahippocampal activation was larger during Upregulation compared to the last half of the preceding baseline phase, a negative value indicates that activation was smaller. Error bars indicate 1 SE. Runs 1–3 were at T2, runs 4–6 at T3, and runs 7–9 on T4.

Analysis of changes in left PHG activation during upregulation phases expressed as PSC yielded no significant results in group HC [*F*(8,112) = 0.992, *p* = 0.446, $\eta_{G}^{2}$ = 0.017]. The same is true for groups PA [*F*(8,72) = 1.624, *p* = 0.133, $\eta_{G}^{2}$ = 0.065] and SH [*F*(8,24) = 0.392, *p* = 0.914, $\eta_{G}^{2}$ = 0.112]. When comparing differences between groups, a trend was found [*F*(2,26) = 2.954, *p* = 0.070, $\eta_{G}^{2}$ = 0.132] indicating HC > PA > SH. No differences over time [*F*(8,208) = 1.246, *p* = 0.274, $\eta_{G}^{2}$ = 0.016] and no interaction between group and time [*F*(16,208) = 1.106, *p* = 0.351, $\eta_{G}^{2}$ = 0.028] were revealed. See Figure 4 for a visualisation of PSC in upregulation phases over time in all groups.

### Granger-Causality-Analysis

Based on functional results, 11 clusters were selected for GCA: right precuneus, left posterior cingulate, left and right PHG, left and right middle frontal gyrus, left and right superior occipital gyrus, left superior temporal gyrus (temporo-parietal-junction), right cerebellar tonsil and right anterior cerebellar lobe.

**Figure 5:**
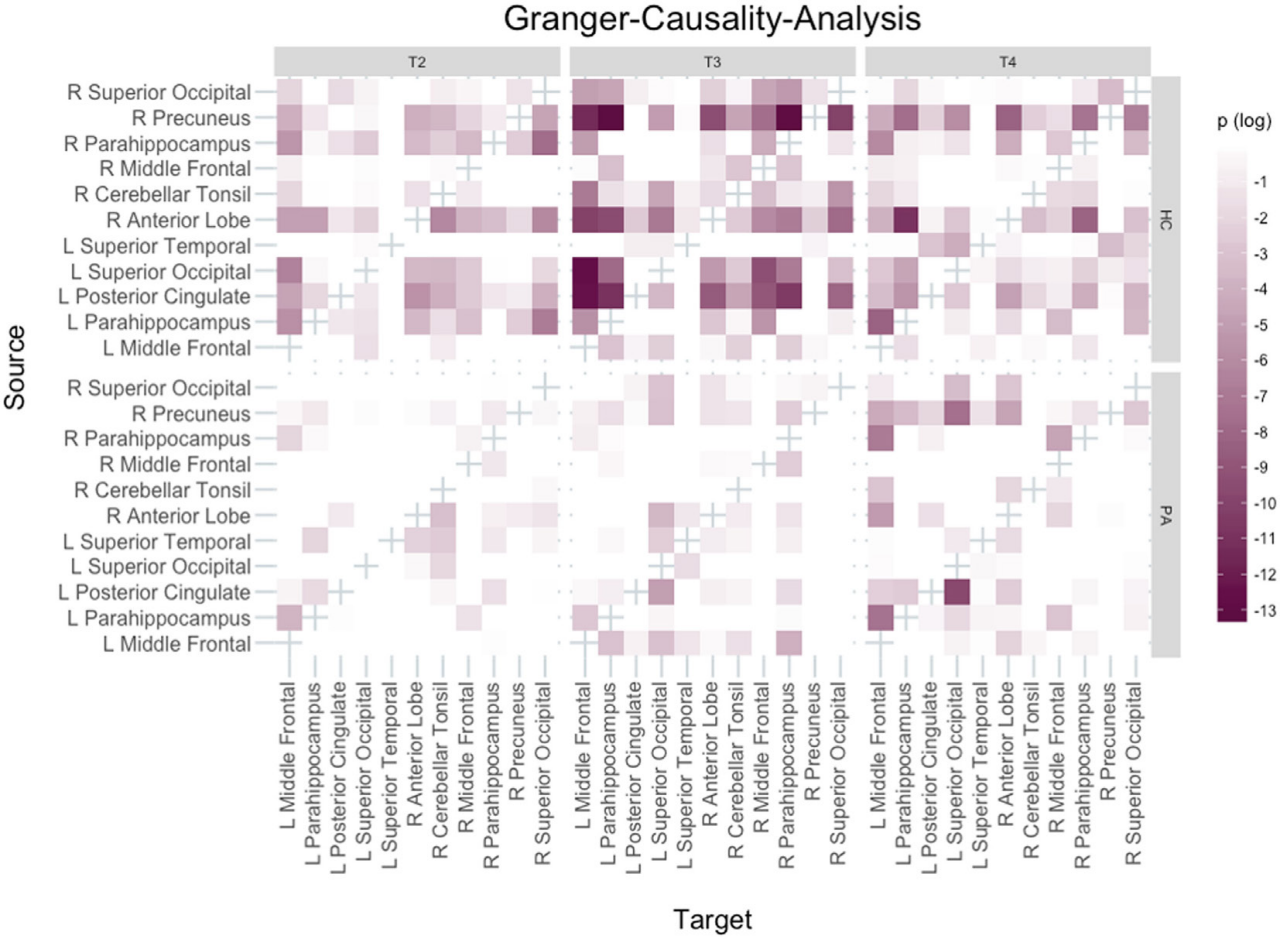
Granger-Causality-Analysis results. Directionality of Granger-causality is from source to target region. Bonferroni–Holm adjusted *p*-values were logarithmised with base 10, i.e., the darker a tile the smaller the *p*-value.

The networks are visualised in Figure 5. The most important findings are that in HC the left PHG appears to change its role to a mainly receiving, but less driving region with an increasing number of inputs and a decreasing number of outputs over the course of the training. In contrast the opposite pattern can be observed for the right precuneus in HC, which becomes mainly a driver of activation.

Similar results were found for group PA regarding the right precuneus, but different than in HC the role of the left PHG remained unchanged across all three training days.

### Voxel-Based Morphometry

Region of interest-based analysis revealed significant GM volume loss at T1 in PA compared to HC in the left PHG [one-sided *t*-test: *t*(16.095) = 2.110, *p* = 0.025] and a trend for the left hippocampus [*t*(10.663) = 1.508, *p* = 0.080]. Nonetheless, voxel-wise comparison of initial GM volumes between groups HC and PA did not yield any significant differences across the whole brain.

When comparing changes over time increases in GM volumes across both groups were found in two locations: the right precuneus (541 vx; *T* = 4.77, *p* = 0.038; MNI 6/−56/21, TAL approx. 6/−53/22) and the right superior medial frontal gyrus (261vx; *T* = 5.87, *p* = 0.048; MNI 9/30/40, TAL approx. 9/31/35). Further trends were found for the middle occipital gyrus (349vx; *T* = 4.85, *p* = 0.052; MNI 36/−76/21, TAL approx. 36/−73/23) and the middle frontal gyrus (163vx; *T* = 5.91, *p* = 0.074; MNI 38/16/54, TAL approx. 38/18/49). Neither decreases, nor interactions between experimental groups and time were found.

### Debriefing after rtfMRI Neurofeedback Training

Group HC had an overall median debriefing score of 2 (MAD = 0). This was lower for group PA with a median of 1 (MAD = 0.5) and group SH with a median score of 1 (MAD = 0.5). The analysis of the subjective rating over the course of time did not suggest any changes for group HC [χ^2^(8) = 4.188, *p* = 0.840]; neither for group PA [χ^2^(8) = 4.636, *p* = 0.769]; nor for group SH [χ^2^(8) = 2.222, *p* = 0.973].

A comparison between all three groups revealed significant differences, though [χ^2^(2) = 12.684, *p* = 0.002]. Pairwise Kruskal–Wallis tests showed that this effect was caused by larger scores in group HC compared to each of the other two groups [HC vs. PA: χ^2^(1) = 9.561, *p* = 0.002; HC vs. SH: χ^2^(1) = 6.436, *p* = 0.011]. Scores between PA and SH were not different [χ^2^(1) = 0.545, *p* = 0.461].

## Discussion

The present study applied rtfMRI neurofeedback training targeting the left PHG and visuospatial memory in healthy elderly and patients of pAD with the aim to improve cognitive performance. Results indicate that the training improved the targeting domain of visuospatial memory in elderly as well as patients. However, in pAD improvement in the target domain was accompanied by decreased performance in a visual memory task. A small group receiving feedback of a brain region unrelated to the paradigm, but otherwise performing the same task, did not display any changes in cognitive performance from pre to post-test. In the healthy control group and in pAD patients GCA suggested changes in networks affecting the parahippocampus and precuneus. Voxel-based morphometry showed structural changes in the precuneus, frontal and occipital areas after training. Generally, this proof-of-concept study is in line with previous rtfMRI neurofeedback training studies (17), but extending the literature with evidence for feasibility and possibly successful application of rtfMRI neurofeedback training to patients with pAD.

The strongest indicator that the conducted training led to the intended outcome is the result of the VVM visuospatial memory test. This measure represents cognitive abilities of the targeted domain; therein we found improvements in groups HC and PA but not in SH. Presence of effects only for groups that underwent training of the PHG suggests that mental imagery alone is insufficient to elicit these effects, and only reliable feedback of the PHG leads to improvements. The concept of mental imagery training alone inducing effects is not novel and paradigms with positive outcomes after mental imagery training have for example been reported in various settings [see, for example, Ref. (47–49)].

The course of parahippocampal activation throughout the training did not reflect behavioural changes in test performance over time. For group HC, PSC of PHG activation during upregulation phases remained stable over the course of the training. For SH PSC of PHG was stable as well, but negative indicating less activation during upregulation than baseline. This fits the unrelated target region and lack of behavioural changes well. While failing statistical significance, patterns of PSC were different for group PA which (1) showed overall lower PSC than group HC, which is in line with findings of decreased memory-related activation in AD in that region (50); (2) despite overall low PSC showed a strong—but not statistically significant—peak on the first feedback run on T3 and (3) displayed mainly negative PSC on T4 suggesting difficulties to perform the task on that day. It remains unclear, why the course of PSC was fluctuating this much in group PA. However, the lower PSC levels compared to HC and the apparent problems in properly performing the task on the last training day seem to fit the fewer increases in behavioural performance in this group compared to HC.

The literature is inconclusive regarding whether an increase or a decrease in brain activation is related to increased cognitive performance. On the one hand, increased activation is often interpreted as translating into increased performance (12, 51); on the other hand decreased activation is usually being explained as increased efficiency also translating into improved function (52, 53). Given the lack of significant changes in PSC in groups HC and PA it seems more plausible to assume that the training lead to increased efficiency. A critical point when looking at previous rtfMRI neurofeedback training studies is that many studies reported a clear trend of activation in the target region over the course of the training [see for example (20, 23, 54)]. Future studies should shed light on the circumstances under which an increase in PSC during the target condition is established and under which circumstances it remains constant.

For all neuropsychological tests beyond the visuospatial memory task of the VVM for which improvements were found in HC, evidence has been reported that the cognitive domains these tests measure are related to the PHG. For working memory, as measured by the backward digit-span task, this is true in cases in which novel information is kept in working memory (55–57), but not in other cases (58, 59). It is certainly debatable whether the task is novel enough to elicit PHG involvement, but given the present results it appears plausible. The issue of PHG involvement is clearer for the MoCA, as many of its subtasks have been reported as being related to the PHG. As detailed above, the task on delayed recall and visuospatial processing target domains associated with the PHG. Furthermore, associations between the PHG and verbal abstraction tasks have also been reported in the literature (60).

The issue of worse performance in the WMS visual memory task in group PA is more difficult to explain. The adoption of techniques and strategies that enhance performance in one cognitive domain but impair another, suggesting negative transfer (61, 62), has been recently reported (63). There is not much further literature on this matter, especially not in the context of rtfMRI neurofeedback training.

Functional MRI and VBM results generally fit the overall concept of the training task and its demands. Activation that was found during upregulation phases represents visuo-motor imagery and retrieval of episodic memory contents well. The substantially less activation that was found in group PA compared to HC, can be well explained by the expected difficulties in recall of memories in group PA. There is conflicting evidence regarding the overall change of activation in AD (64), so it is difficult to conclude whether the activation found in group PA is due to difficulties in performing the task or due to the effects of pAD. As expected, no PHG activation and overall low activation was found in SH. A critical point here may be the small sample size making broad activation less likely to be found, but the results of the PSC analysis show that activation was indeed lower during upregulation phases than during baseline in group SH. A possibility here may be that generation of numerous different strategies for modulation of left PHG activation due to the unresponsive feedback stimulus hampers broad activation and consequently enhanced cognitive performance.

Differences in GM volume between groups were found in the left hippocampus and parahippocampus. These are regions associated with AD and a loss of GM in PA in these regions thus expected. The lack of a group difference in the whole-brain analysis seems unusual, though, but is not implausible given both the prodromal state of the disease and the small group size. Longitudinally on the whole-brain level, while no changes in left PHG volume were found, the finding of increased GM volume in the right precuneus fits well into the data. Large clusters of activation were found for that region during upregulation phases of rtfMRI neurofeedback training and that region has been associated with memory retrieval, visuo-motor imagery and aspects of consciousness (65, 66). It has also been hypothesised that the parietal lobe and especially the precuneus might be critical for AD (67, 68) suggesting that for the conducted training the precuneus is an essential region and a GM volume increase therein is especially promising for AD. Moreover, increased GM volume in the middle occipital gyrus fits well into the present paradigm, as a core component of it was mental imagery of a previously memorised real-world footpath. Finally, also the findings for both frontal regions can be explained well in the context of the training. The medial location has been associated with cognitive control (69, 70) which is a cognitive component required to monitor the state of the feedback stimulus and to adapt strategies accordingly. The middle frontal location is in contrast associated with working memory (71), a cognitive component which is very relevant for the training task as the state of the feedback stimulus not only needs to be monitored, but also compared to previous states, to successfully apply modulation strategies. Effects on GM volume induced by cognitive training over the course of a few weeks have been demonstrated recently (72).

Granger-causality-analysis results suggest that both in group HC and group PA the precuneus becomes the main driver of activation. There are some reports of the precuneus playing a role for feedback learning (73, 74), but none in the context of a paradigm comparable to the present study. The possible involvement of the precuneus in feedback learning, together with its associations elaborated on above, seems plausible to explain its changed role here. Together with the VBM results this points out the importance of this region for this kind of training. The role of the target region is also very interesting and differing between groups. In group HC, the left PHG seems to become less of a driver of activation over the course of the training, while in group PA it does neither receive many inputs nor is it the source of many connections.

It should be noted that the application of GCA to fMRI data has been subject of debate (75, 76). Main points raised against the application of GCA to fMRI data are (1) the low temporal resolution of such data leading to interactions between regions impossible to be displayed; and (2) the fact that the haemodynamic response latency differs between regions of the brain and thus might confound data. There is evidence though, that given met prerequisites and properly formulated research questions GCA on fMRI can provide useful results (77, 78). Nevertheless it should be kept in mind that connections faster than the selected lag size are missed entirely. General issues of correlative analyses such as the problem of potential third variables affecting the data apply as well.

As control condition we used a sham-feedback approach with the presentation of feedback from a brain region unrelated to the task (79). A problem with this approach is that subjects may find the strategies they apply have no influence whatsoever on the feedback and thus become frustrated and demotivated. On the one hand, the finding of no cognitive improvement in group SH in the present work is evidence for the effectiveness of the training paradigm, on the other hand caution is advised in the interpretation of the results as the performance in group SH may have been negatively influenced by motivation, which is known to moderate cognitive training outcome (80). Future research should aim to develop control conditions that explicitly target the motivational problem.

Difficulties in influencing the feedback stimulus are also a concern for the patient group. Subjective ratings of the ability to influence the feedback thermometer indicate that subjects of group PA faced similar difficulties as group SH despite receiving true feedback of the PHG, implying the same considerations regarding motivation. As it has been suggested previously that a certain intactness of cognitive functioning is required for successful applications of neurofeedback and brain–computer interface paradigms (81), such implementations in the future should be applied as early as possible in the course of the disease to ensure optimal results.

### Limitations

This study has some limitations. First, although the spatial navigation task was standardised as much as possible, it took place in a dynamic, constantly changing real-world environment leading to slight differences in paths between subjects. Another limitation is the small sample size and reduced power of analyses in group SH, so subtle effects could not be detected and the interpretability of neuropsychological results from this group is rather limited. Finally, no follow-up assessment has been done to test whether observed effects persist. There are reports of EEG-neurofeedback effects being present after months to years after the initial training (82–85). Not much work has been done in this regard for fMRI-based neurofeedback [examples of studies with follow-up term-assessments are Ref. (19, 86, 87)].

## Conclusion

In summary, this proof-of-concept study suggests that rtfMRI neurofeedback training paradigms are feasible in patients of pAD and in the healthy elderly and may be used to counteract cognitive effects of both AD and healthy ageing. As a small group of healthy subjects receiving sham-feedback only did not display PHG activation or cognitive changes over time, it is concluded that mental imagery alone is insufficient to achieve the effects observed in the groups receiving feedback relevant to the task. Neurofeedback training yielded an activation of the target region PHG in elderly and pAD, which was associated with changes of connectivity and GM volume. Future research needs to address the issues of task difficulty in AD patients and of potential transfer effects on untrained cognitive domains. Overall, our results are promising regarding future clinical applications of rtfMRI neurofeedback training as a tool to slow down cognitive decline.

## Ethics Statement

After full introduction of the study, all subjects gave written informed consent in accordance with the Declaration of Helsinki. The protocol was approved by the local institutional review board at RWTH Aachen University (local reference number EK 049/11).

## Author Contributions

CH took part in recruiting subjects and acquiring data, carrying out data analysis except Voxel-Based-Morphometry, interpreting the data, and writing of the manuscript. NN, HK, and SK took part in recruiting subjects and acquiring data as well as designing the study. ID carried out Voxel-Based-Morphometry, wrote the sections on Voxel-Based-Morphometry of the manuscript, and revised the first draft of the manuscript. CM took part in recruiting subjects and acquiring data. FP contributed to data acquisition and Voxel-Based-Morphometry. RG and AH contributed to setting up the real-time fMRI paradigm and provided assistance with fMRI analysis. NS contributed to the design of the study and provided MR infrastructure. JS contributed to the design of the study. MR took part in study design, data interpretation, and revised the first draft of the manuscript. KR contributed to the design of the study, acquired funding, facilitated recruitment of patients, took part in interpretation of data, and revised the drafts of the manuscript. All authors read and approved the final manuscript.

## Conflict of Interest Statement

The authors declare that the research was conducted in the absence of any commercial or financial relationships that could be construed as a potential conflict of interest.
